# Differential expression profile of microRNAs associated with human breast cancer progression

**DOI:** 10.1186/1753-6561-7-S2-P9

**Published:** 2013-04-04

**Authors:** Augusto LF Marino, Adriane F Evangelista, Taciane Macedo, Henrique CS Silveira, Ligia M Kerr, Rene AC Vieira, Adhemar F Longatto, Márcia MCM Silveira

**Affiliations:** 1 Molecular Oncology Research Center, Barretos Cancer Hospital, Barretos, SP, Brazil; 2 Department of Pathology, Barretos Cancer Hospital, Barretos, SP, Brazil; 3 Department of Mastology and Breast Reconstruction, Barretos Cancer Hospital, Barretos, SP, Brazil

## Background

MicroRNAs (miRNAs) negatively regulate gene expression and its deregulation is involved in cancer progression. Our aim was to verify which miRNAs may play a role in breast cancer progression, especially metastasis.

## Materials and methods

MicroRNA expression profiles were generated by microarray analysis (Agilent) of 70 samples from primary breast tumors with different clinical staging. Data were analyzed using bioinformatics software (R, Cluster and Tree View), which allowed sample discrimination (metastatic *vs*. non metastatic tumors) by hierarchical clustering of breast cancer expression signatures. We performed a second analysis using the ROC curve method that allowed us to identify miRNAs with greater predictive potential of malignancy. Furthermore, by Venn diagram analysis, we were able to identify 8 miRNAs that were differentially expressed in all metastatic tumors of different clinical staging, compared to the primary non-metastatic tumors. SPSS 19 software was used to generate a Kaplan-Meier curve (p<0.05) to estimate the disease-specific survival (considering the specific event such as death by cancer), based on miRNA expression patterns.

## Results

We found four miRNAs previously described as potentially oncogenic (hsa-let-7a, hsa-let-7b, hsa-let-7c and hsa-miR-21) for breast cancer, being hsa-let7a, hsa-let7b and hsa-miR-21 under-expressed in the metastatic vs non-metastatic tumors, and four new miRNAs candidates for markers of metastasis (has-miR-1308, has-miR-923, hsa-miR-328, has-miR-494), the first two were over-expressed while the latest ones were under-expressed in metastatic vs non-metastatic tumors.

## Conclusions

We identified two groups of miRNAs whose expression level were associated with worse prognosis. These findings are important for better understanding of the role of miRNAs in breast cancer progression.

## Financial support

FAPESP.

